# Phylogenomic analysis reveals persistence of gonococcal strains with reduced-susceptibility to extended-spectrum cephalosporins and mosaic *penA*-34

**DOI:** 10.1038/s41467-021-24072-1

**Published:** 2021-06-21

**Authors:** Jesse C. Thomas, Sandeep J. Joseph, John C. Cartee, Cau D. Pham, Matthew W. Schmerer, Karen Schlanger, Sancta B. St. Cyr, Ellen N. Kersh, Brian H. Raphael, the Antimicrobial Resistant Neisseria gonorrhoeae Working Group, Cathy Dominguez, Ami Patel, Jillian Loomis, Sopheay Hun, Ryan Ruiz, Nicole Talosig, Chi Hua, Jenny Zhang, Bonnie Oh, John Leavitt, Christina Moore, Zach Perry

**Affiliations:** 1 Division of STD Prevention, Centers for Disease Control and Prevention, Atlanta, GA USA; 2 Maryland Department of Health, Maryland Regional Lab, Baltimore, MD USA; 3 Washington State Department of Health, Washington State Regional Lab, Shoreline, WA USA; 4 Texas Department of State Health Services, Texas Regional Lab Austin, Austin, TX USA; 5 Tennessee Department of Health, Tennessee Regional Lab Nashville, Nashville, TN USA

**Keywords:** Phylogenetics, Bacterial genetics, Urogenital diseases, Epidemiology

## Abstract

The recent emergence of strains of *Neisseria gonorrhoeae* associated with treatment failures to ceftriaxone, the foundation of current treatment options, has raised concerns over a future of untreatable gonorrhea. Current global data on gonococcal strains suggest that several lineages, predominately characterized by mosaic *penA* alleles, are associated with elevated minimum inhibitory concentrations (MICs) to extended spectrum cephalosporins (ESCs). Here we report on whole genome sequences of 813 *N. gonorrhoeae* isolates collected through the Gonococcal Isolate Surveillance Project in the United States. Phylogenomic analysis revealed that one persisting lineage (Clade A, multi-locus sequence type [MLST] ST1901) with mosaic *penA-34* alleles, contained the majority of isolates with elevated MICs to ESCs. We provide evidence that an ancestor to the globally circulating MLST ST1901 clones potentially emerged around the early to mid-20th century (1944, credibility intervals [CI]: 1935–1953), predating the introduction of cephalosporins, but coinciding with the use of penicillin. Such results indicate that drugs with novel mechanisms of action are needed as these strains continue to persist and disseminate globally.

## Abstract (web summary)

Resistance of Neisseria gonorrhoeae to extended spectrum cephalosporins is an increasing concern. Here, the authors conduct whole genome sequencing of isolates from the United States and find that most resistant isolates were associated with a persistent circulating lineage.

## Introduction

*Neisseria gonorrhoeae* gonorrhoeae, the causative agent of the sexually transmitted infection gonorrhea, was ranked as one of the most “urgent” threats in the Centers for Disease Control and Prevention’s (CDC) Antibiotic Resistance Threats in the United States 2019 report^1^. Gonorrhea is the second most reported infectious condition in the United States (583,405 cases were reported to CDC in 2018) with an estimated yearly incidence of 87 million infections worldwide^2,3^. Historically, beginning with sulfonamides in the late 1930s, the gonococcus has successively developed resistance to all previously recommended antimicrobial drugs used for treatment of gonorrhea (e.g., penicillins, tetracyclines, macrolides, fluoroquinolones, and extended-spectrum cephalosporins [ESCs])^4^. This led to several countries including the United States in 2010 to recommend combination therapy for gonorrhea treatment as it was theorized that other co-administered agents (e.g., doxycycline or azithromycin) would delay the emergence and dissemination of cephalosporin-resistant gonococcal strains^5^. Following the worldwide rise in cefixime resistance, treatment guidelines changed, and ceftriaxone became the cornerstone of therapy^6,7^. Beginning in 2015, doxycycline was removed as part of combination therapy leaving ceftriaxone (250 mg injected intramuscularly) and azithromycin (1 g taken orally) as the only recommended therapy for uncomplicated gonorrhea^8^. In 2020, combination therapy was no longer recommended and ceftriaxone (500 mg, 1 g for persons weighing ≥150 kg) became the only recommended regimen for uncomplicated gonorrhea^9^.

The gonococcus has adapted a myriad of strategies enabling it to survive within different niches of the human host and in the presence of antimicrobials, including the use of (i) efflux pumps, (ii) target modification of antimicrobial substrates, (iii) reduction of outer membrane permeability, among others^10,11^. The capacity of gonococci to develop antimicrobial resistance (AMR) is largely attributed to horizontal gene transfer or specific mutations^4^. The gonococcus is naturally competent for genetic transformation and can import DNA from extracellular sources into its genome by homologous recombination^10^. Commensal *Neisseria* species (e.g., *N. cinerea*, *N. flavescens*, *N. perflava*, etc.) and even other gonococci that share the same host niche can be reservoirs of this extracellular DNA^10,12,13^. Reduced susceptibility to ESCs has been primarily associated with an altered *penA* gene encoding penicillin-binding protein (PBP) 2^4^. PBP2 is an important transpeptidase (TPase) among a collection of four PBPs including: PBP1 (*ponA*; TPase), PBP3, and PBP4 (carboxypeptidase/endopeptidase) necessary for biosynthesis of peptidoglycan, the main component of the cell wall^14,15^. All β-lactams antimicrobials (e.g., penicillins, cephalosporins, carbapenems, and carbacephems) exert their lethal action by binding and inhibiting PBPs that are necessary for inserting cross-linkage structures into the cell wall^10,14–16^. PBP2 is particularly important as it is the main lethal target of β-lactam antibiotics used for treatment of gonorrhea^10,16^. By the 1980s, studies had noted penicillin-resistant strains with altered forms of PBP2 characterized by an Asp-345 insertion and other mutations, which was later attributed to interspecies recombination with *Neisseria* commensals^17,18^.

As early as the 2000s, gonococcal strains were observed with reduced susceptibility to oral cephalosporins (e.g., cefixime, cefdinir, cefpodoxime, cefepime, etc.) in Japanese metropolitan areas, where their usage was common^19–22^. These isolates were shown to harbor “mosaic” *penA* alleles, which can contain 60–70 amino acid changes in PBP2 compared to a wildtype (WT) PBP2 sequence, and originate from homologous recombination with *Neisseria* commensals^7,10,23,24^. Some of the earliest isolates with reduced susceptibility to cefixime were collected from Japan in the 1990s–2000s and contained mosaic *penA*-10^7,19,22,24^. In 2009, the first mosaic *penA*-34 (≥98% sequence homology to *penA*-10) harboring isolates with reduced susceptibility to cefixime were described in the United States, although recent whole genome sequencing (WGS) and AMR determinant screening suggest that these strains had emerged as early as 2006^25,26^. Currently, strains with mosaic *penA*-34 and its derivatives (mosaic alleles sharing ≥98% sequence homology) are prevalent in the United States and globally^27–30^. In addition, certain amino acid substitutions in the mosaic *penA* genes observed in gonococcal strains such as WHO-Y (*penA*-44; A501P), H041 (*penA*-37, A311V, A316P, and T483S), and clones of strain FC428 (*penA*-60; A311V and T483S) have been shown to confer high-level cefixime (CFM^HL^, minimum inhibitory concentrations [MICs] ≥1 µg/mL) and ceftriaxone MICs (CRO^HL^, MICs ≥0.5 µg/mL)^6,31,32^. Compensatory changes in secondary loci such as the *acnB* gene have also been shown to increase bacterial in vitro fitness in isolates with mosaic *penA* alleles, which have been demonstrated to impart a biological fitness cost^33^. Additional pathways for resistance to β-lactams include the acquisition of multiple chromosomal mutations that additively increase resistance: mutations in outer membrane porins (PorB_1B_), in the PilQ secretin of type IV pili, and in *mtr*^4,33–39^.

In the United States, sentinel surveillance of *N. gonorrhoeae* is conducted through CDC’s Gonococcal Isolate Surveillance Project (GISP). Notably, GISP includes a system of sexually transmitted disease (STD) clinics that collects urethral specimens for culture and determines the antibiotic MICs to selected drugs, currently or previously used to treat gonorrhea, each month from the first 25 male patients with gonococcal urethritis^40^. GISP uses MIC values to monitor trends in antimicrobial susceptibility over time^41^. Based on GISP data, the percentage of isolates with elevated cefixime MICs (CFM^em^; MIC ≥0.25 µg/mL) peaked at 1.4% nationally before CDC updated its treatment recommendations in 2012 but has since fluctuated around 0.4% between 2013 and 2018^41^. In addition, the percentage of GISP isolates that exhibited elevated ceftriaxone MICs (CRO^em^; MIC ≥ 0.125 µg/mL) has fluctuated around 0.2% between 2012 and 2018^41^.

In this observational study, we examined the genomic diversity and AMR determinants of circulating *N. gonorrhoeae* lineages collected by GISP in the United States between 2005 and 2017. We were primarily interested in how gonococcal susceptibility patterns to ESCs have emerged over time in the United States, and how such strains have evolved and acquired mutations in *penA* following previous reports^26,28,29^. In addition, we conducted a global phylogenomic comparison on a subset of isolates with elevated cephalosporin MICs collected from the United States and other countries. From this, we assessed the genetic similarity of mosaic *penA* containing *N. gonorrhoeae* strains and estimated their likely emergence and spread.

## Results

### Strain diversity and distribution

A variety of diverse sequence types (STs) were detected from the 813 isolates. Ninety multi-locus sequencing typing (MLST) STs were identified, with the majority of isolates belonging to five major STs including ST1901 (38%, 308/813), ST9363 (11%, 87/813), ST7363 (5%, 40/813), ST1580 (4%, 32/813), and ST1584 (3%, 27/813). Isolates belonging to the ST1901 (71%, 235/332; *P* < 0.0001) and ST1580 (10%, 30/332; *P* < 0.0001) groups were significantly associated with CFM^em^; however, only those from the ST1901 were significantly associated with CRO^em^ (75%, 75/104; *P* < 0.0001). Furthermore, isolates were assigned to 198 unique *N. gonorrhoeae*multi-antigen sequencing typing (NG-MAST) STs, which differentiated many of the broader MLST ST groups (MLST STs differing by 1 allele). For instance, ST1407 (18%, 148/813), the largest group of NG-MAST STs, was observed among isolates with MLST STs 1901, 10312, 9365, and 7360. Isolates with NG-MAST ST3158 were detected only among MLST ST1901 strains, whereas NG-MAST 3935 was only detected among MLST STs 9363 and 11422 strains. In terms of antimicrobial susceptibility, isolates belonging to the NG-MAST ST1407 (41%, 137/332; *P* < 0.0001) and ST1580 (9%, 30/332; *P* < 0.0001) groups were significantly associated with CFM^em^; however, there were no clear significant associations observed between this ST or others with respect to CRO^em^.

### Phylogenomic analysis of circulating lineages with elevated cephalosporin MICs in the United States

**Fig. 1:**
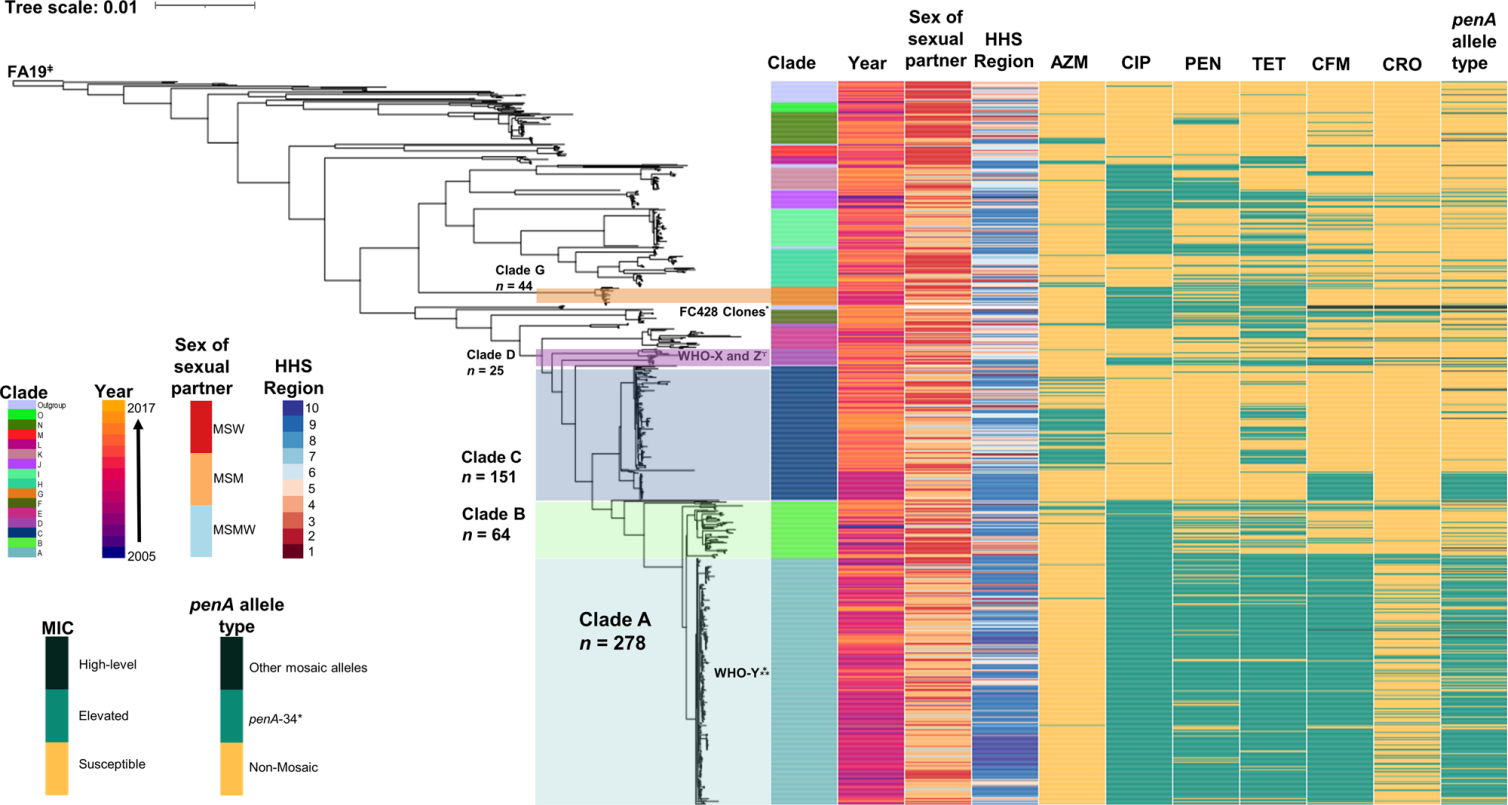
Maximum likelihood tree based on core genome SNPs of 813 *Neisseria gonorrhoeae* strains collected from 2005 to 2017 in the United States, including clade/fastbaps cluster, year, and sex of sex partner. MSW men that have sex with women, MSM men that have sex with men, MSMW men that have sex with men and women, HHS Health and Human Services region, MIC minimum inhibitory concentration with respect to azithromycin (AZM), ciprofloxacin (CIP), penicillin (PEN), tetracycline (TET), cefixime (CFM), and ceftriaxone (CRO). Highlighted clades contain isolates where we identified the majority of elevated cephalosporin MICs. The tree also includes several reference genomes: FA19 (root, ǂ), international MLST ST1903 strains possessing the mosaic *penA*-60 allele (*) including strains from Japan (FC428, FC460) and Canada (47707); WHO reference strains WHO-X (H041, Japan) and WHO-Z (A8806, Australia) marked by (ɤ); and WHO-Y (F89, France) marked by (⁂). A summary of antimicrobial susceptibility to each respective antibiotic is shown with colors: susceptible (light orange), elevated MIC range (teal), or high-level MICs (dark green). Alleles for *penA* are colored based on their amino acid sequence homology to *penA*-34 (≥98% is a derivative allele; teal), differing mosaic *penA* allele (dark green), or absence of a mosaic *penA* allele.

After detection and removal of putative recombinant sites using Gubbins, the number of polymorphic sites estimated to be present in the non-recombinant part of the whole genome alignment was 25,140 nucleotides (nts)^42^. The overall recombination to mutation ratio (r/m) was 2.3, calculated as the ratio of base substitutions predicted within the predicted areas of recombination to those occurring through point mutations, and is similar to other previously reported studies^26,43,44^. The overall percentage of bases under clonal frame was approximately 71%. Fastbaps partitioned the maximum likelihood (ML) phylogeny into 15 clades (A–O, Fig. 1). In total there were seven clades that contained at least two GISP isolates with elevated cefixime and/or ceftriaxone MICs including clades A through G. Both the reference strain FA19 and international MLST ST1903 strains possessing the mosaic *penA*-60 allele (strains A7846, A7536, FC428, FC460, 47707) appeared in separate lineages as distant outgroups^45,46^. Reference strains WHO-X and WHO-Z clustered with several GISP isolates in Clade D, but were distantly related^45^. The reference strain WHO-Y clustered with several GISP isolates with elevated cephalosporin MICs in Clade A. A summary of elevated MIC frequency, MLST STs, and intra-clade SNP distances observed in major clades of interest is presented in Table 1.

**Table 1:** Summary of clades identified in dataset including intra-clade single nucleotide polymorphisms (SNP) distances, total counts of isolates and those with elevated cefixime (CFM) or ceftriaxone (CRO) minimum inhibitory concentrations (MICs), collection year, and predominant multi-locus sequence type (MLST).

No. of isolates with elevated MIC						
Clade	SNP distance, mean ± SD	No. of isolates within clade	CFM	CRO	Isolate collection year	Predominant MLST
Clade A	60.38 ± 28.55	278	259	63	2006–2017	1901
Clade B	351.75 ± 202.12	64	11	11	2005–2017	1901
Clade C	163.81 ± 112.1	151	32	0	2009–2016	9363
Clade D	902.06 ± 508.36	25	6	4	2012–2017	7822
Clade E	428.4 ± 161.29	23	4	2	2006–2017	1893
Clade F	100.47 ± 79.75	15	3	8	2014–2017	7827
Clade G	606.62 ± 325.5	44	10	6	2005–2017	1579
Clade H	86.06 ± 127.66	42	1	2	2013–2017	7363
Clade I	152.17 ± 101.66	25	1	0	2015–2016	5624
Clade J	80.52 ± 47.57	20	3	5	2006–2016	1901
Clade K	112.17 ± 76.83	21	1	1	2011–2015	1588
Clade L	256.22 ± 231.76	9	0	0	2011–2016	10,932
Clade M	222.03 ± 141.08	12	1	0	2012–2015	10,931
Clade N	300.99 ± 226.17	36	0	0	2006–2016	1584
Clade O	443.16 ± 247.37	11	0	0	2011–2014	1585

Clade A (*n* = 278), the largest clade observed in the dataset, was comprised of a diverse collection of isolates spanning several Health and Human Services (HHS) regions (Fig. 1). This clade includes 84 isolates that were first reported by Grad et al.^26^ as the “cef^RS^ cluster 1” in addition to many others collected by GISP between 2006 and 2017. Clade A was represented predominately by MLST ST1901 (87%, 242/278; *P* < 0.0001) and NG-MAST ST1407 (53%, 148/278; *P* < 0.0001), and displayed significant associations with CFM^em^ (78%, 259/332; *P* < 0.0001) with most isolates possessing CFM MICs of 0.25 µg/mL (*n* = 246). This clade also contained the bulk of CRO^em^ isolates in the dataset (61%; 63/104; *P* < 0.0001) with most possessing CRO MICs of 0.125 µg/mL (*n* = 60). Isolates in this clade were characterized by the presence of a variety of mutations in loci commonly associated with elevated cephalosporin MIC, such as the presence of an adenine [A] deletion in the *mtrR* promoter in combination with the H105Y mutation in the *mtrR* coding region (CFM^em^: 75%, 250/332; CRO^em^: 58%, 60/104; *P* < 0.0001). The majority of CFM^em^ isolates possessed a mosaic *penA*-34 allele containing the key amino acid mutations I312M, V316T, N512Y, and G545S (77%, 254/332; *P* < 0.0001), although two isolates possessed a mosaic *penA*-72 (*penA*-34 + P551 mutation)^47^. The *penA*-34 (58%, 60/104; *P* < 0.0001) and *penA-*72 allele (*n* = 1) were also observed in CRO^em^ isolates. In addition, most isolates with elevated cephalosporin MICs in this clade also possessed the L421P mutation in PonA (94%, 260/278; *P* < 0.0001), which overall was significantly associated with CFM^em^ (89%, 297/332; *P* < 0.0001) and CRO^em^ (99%, 103/104; *P* < 0.0001). We detected double mutations at amino acid residues Gly-120 and Ala-121 of PorB in the majority of the Clade A isolates with elevated cephalosporin MICs (256/278; *P* < 0.001), which was also significantly associated with CFM^em^ (85%, 283/332; *P* < 0.001) and CRO^em^ (95%, 99/104; *P* < 0.0001).

A separate lineage of MLST ST1901 isolates was also observed (Clade B, *n* = 64, years 2005–2017) sharing a common ancestor with those in Clade A, but differed considerably in the diversity of NG-MAST STs and overall SNPs (average difference of >350 SNPs) (Table 1 and Fig. 1). Only eight isolates were detected with either CFM^em^ or CRO^em^, and with the exception of two isolates all possessed a mosaic *penA*-10 allele, which shares 98% homology to *penA*-34, but differs at the C-terminal end (amino acids 549–582)^48^. All CFM^em^ or CRO^em^ isolates possessed an adenine [A] deletion in the *mtrR* promoter, MtrR H105Y mutation, the PonA L421P mutation, and six of the eight possessed the PorB G120K/A121D mutations.

Clade C (*n* = 151) contained a sub-lineage of 30 CFM^em^ isolates (MLST ST1580; NG-MAST STs 5895 and 9604) from years 2009 to 2012, which were all susceptible to ceftriaxone^26^. These isolates were characterized by the presence of a G45D mutation in the MtrR coding region and a mosaic *penA*-34 allele (*n* = 32). All isolates in this clade possessed both a WT *ponA* and *porB* gene. Outside of Clade C and the aforementioned clades, there were 27 CFM^em^ isolates and 28 CRO^em^ isolates that appeared sporadically throughout the phylogenetic tree. These isolates were collected from multiple HHS regions and years (2009–2017), were represented by several STs, and varied in co-occurring mutations across various antimicrobial AMR loci. A closer examination of 14 identified isolates with combined elevated cefixime and ceftriaxone MICs indicated that 7 of the 14 possessed a A-deletion in the MtrR promoter, and 11 possessed a least one or a combination of mutations in the MtrR coding region including the A39T (*n* = 1), G45D (*n* = 7), and H105Y (*n* = 2) mutations. In addition, we also detected six unique PBP2 patterns including those that encode a mosaic *penA* (10, 27, and 34) and non-mosaic *penA* alleles (12, 13, and 68). All 14 isolates possessed a WT PBP1, and nine possessed the G120K/A121D mutations in PorB.

Recently, it was shown in clinical gonococcal isolates without mosaic *penA* alleles that mutations in the RNA polymerase genes (i.e., *rpoB* and *rpoD*) can independently give rise to elevated cephalosporin MICs^49^. Accordingly, we examined our dataset and identified 32 isolates (2009–2017) distributed throughout the phylogeny with elevated cephalosporin MICs (ceftriaxone MICs ≥0.125, cefixime MICs ≥0.25 µg/mL) that could not be explained by mutations in PBP2. A closer look at the WGS data revealed several previously reported mutations in the RNA polymerase genes *rpoB* and *rpoD* (Supplementary Data 1). Fourteen of the isolates possessed a WT *rpoB* gene, while 17 possessed the H553N mutation (Clade B, *n* = 1; Clade D, *n* = 3; Clade F, *n* = 5; Clade G, *n* = 1; Clade H, *n* = 1; Clade J, *n* = 5). We did not detect any additional isolates, aside from three previously reported strains included in this study’s dataset, with either the *rpoB* R201H mutation (Clade E, *n* = 1), deletion of amino acids 92–95 (Clade D, *n* = 1), or the E98K mutation in the *rpoD* gene (Clade D, *n* = 1)^49^. A summary of all unique mutations in the *rpoB* and *rpoD* genes is provided in Supplementary Data 1 and Supplementary Figs 1 and 2.

### Tracing the global emergence of Clade A-B isolates using timed-scaled phylogenetic analysis

**Fig. 2:**
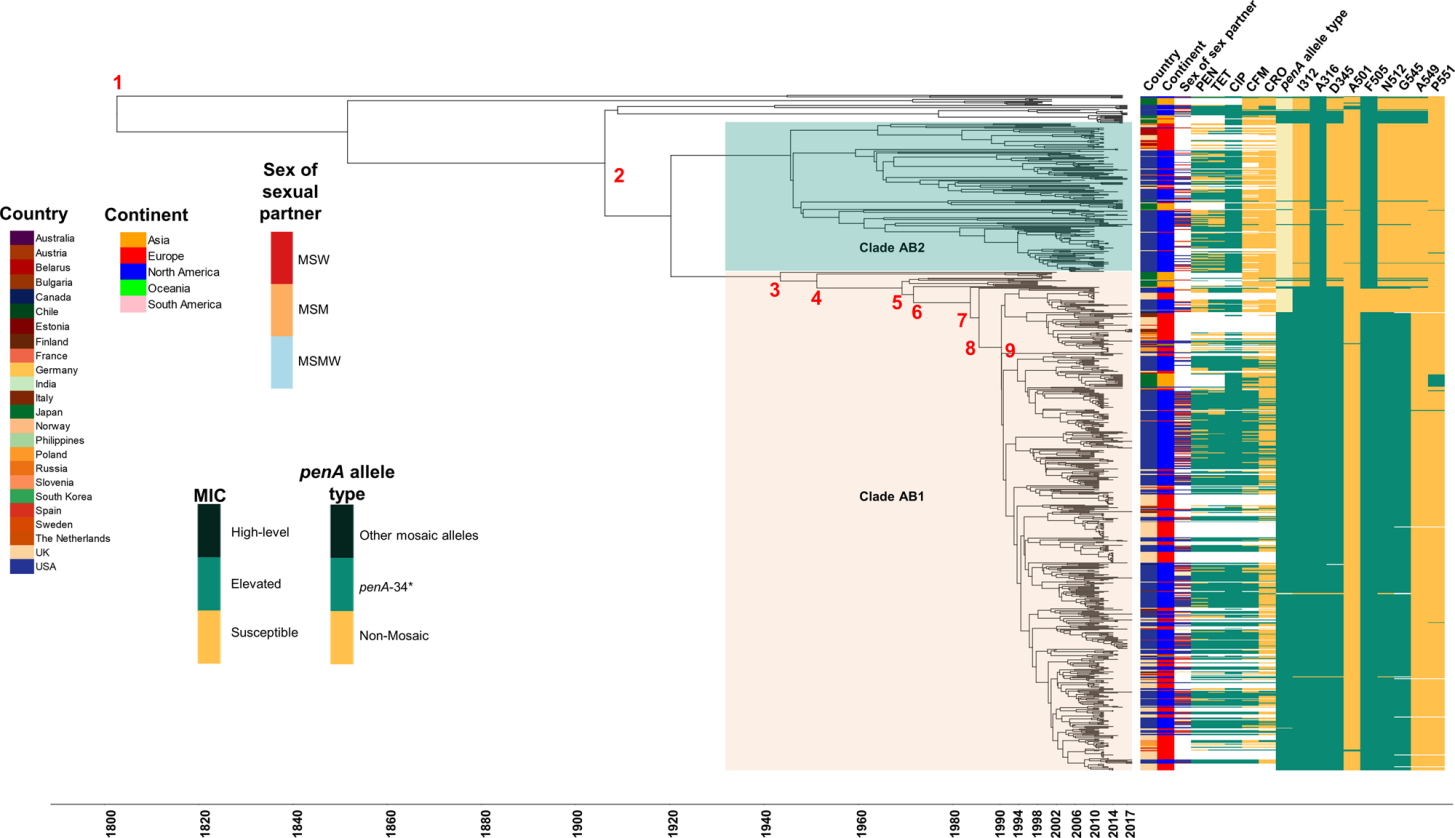
Time-scaled (strict model) BEAST phylogeny of 805 isolates, including 340 GISP isolates identified in Clade A-B of this study and 465 international MLST ST1901 strains. Clade AB1 and Clade AB2 represent the divergent sub-lineages that we identified in the phylogeny. The oldest sequenced isolate is from the Philippines collected in 1992. The phylogeny includes epidemiological details and antimicrobial susceptibility. Sex of sex partners are colored [MSW men that have sex with women (red), MSM men that have sex with men (light orange), MSMW men that have sex with men and women (light blue)]. Antimicrobial susceptibility is based on minimum inhibitory concentration (MIC) to each respective antibiotic and is shown with colors: susceptible (light orange), elevated MIC range (teal), or high-level MICs (dark green). Alleles for *penA* are colored based on their amino acid sequence homology to *penA*-34 (≥98% is a derivative allele; teal), differing mosaic *penA* allele (dark green), or absence of a mosaic *penA* allele. Amino acid mutations in PBP2 associated with elevated cephalosporin MICs are shown at the top of figure. The bottom scale bar represents the time to the most recent common ancestor (tMRCA) of the internal node of the phylogenetic tree.  Numbers highlighted in red are the ancestral nodes of the time-scaled phylogenetic tree and represent estimated years and credibility intervals based on tMRCA: 1) Root; 1818 [1781 - 1865], 2) 1925 [1913 - 1951], 3) 1944 [1935 - 1953], 4) 1952 [1942 - 1964], 5) 1970 [1964 - 1977], 6) 1972 [1966 -1979], 7) 1985 [1980 - 1990], 8) 1987 [1983 - 1992],  9) 1990 [1986 - 1994].

To generate a more detailed understanding of the geographic origin and spread of isolates with elevated cephalosporin MICs (ESC^em^), we performed timed phylogenetic analyses using BEAST, specifically focusing on the Clade A-B isolates (*n* = 340, 278 with the MLST ST1901 designation) where the majority of ESC^em^ was detected. We included an additional 465 international isolates (MLST ST1901), which along with Clade A-B isolates fall within the cgMLST cgc400 core genome group no. 3 (Supplementary Data 1 and 2)^50^. From the BEAST analysis, we estimated the substitution rate for the non-recombinant region of the genome collection at 1.8215 × 10^–6^ substitutions per site per year, (95% highest posterior density (HPD) ranging from 1.62323 × 10^–6^ to 2.069 × 10^–6^) and 2.3638 × 10^–6^ substitutions per site per year (95% HPD; 2.035 × 10^–6^–3.1145 × 10^–6^) for the strict (Fig. 2/Supplementary Table 1) and relaxed (Supplementary Fig. 3/Supplementary Table 2) clock model). The substitution rates are similar to previous estimates from other population-level studies that included *N. gonorrhoeae* strains from a single or similar STs^26,51,52^. For both models, we estimated credibility intervals (CIs) for the most recent common ancestor (tMRCA) in selected internal nodes (Supplementary Table 3). We estimated the tMCRA for the Clade A-B isolates (*n* = 805) to be around the early twentieth century. The clade diverged into two sub-lineages around the 1920s (1925, CI: 1913–1951), which we denoted as Clade AB1 and Clade AB2. The larger Clade AB1 contained the majority of the US isolates (*n* = 594) along with international isolates (*n* = 147), and were estimated to have emerged from a common ancestor that existed in 1944 (95% CI: 1935–1953) (Supplementary Table 3). The smaller Clade AB2 strains are largely susceptible (*n* = 64), but contained a small group of ESC^em^ isolates from North America, Asia, and Europe with mosaic *penA*-71,72, or 75 alleles, all of which appear to have emerged rather sometime around the early 2000s. In addition, while both lineages possess a genomic background with multiple resistances to penicillin, tetracycline, and ciprofloxacin, the majority of ESC^em^, particularly to cefixime, were found in Clade AB1. Interestingly, it is in Clade AB1 where we observed the majority of mosaic *penA* alleles; notably *penA*-34 and similar derivatives.

## Discussion

In light of several reports that have examined the global rise in azithromycin MICs, the importance of monitoring resistance to ESCs, particularly ceftriaxone—the last universally effective drug, cannot be overstated. Recently, in countries such as the United Kingdom and the United States, azithromycin was removed as a co-administered agent from national treatment guidelines in favor of ceftriaxone monotherapy^9,53–58^. We conducted a large study on gonococcal isolates collected by sentinel surveillance in the United States, with a focus on reduced susceptibility to ESCs. Data for this study were collected over a 13-year period from geographically diverse STD clinics and represents a reasonable approximation of circulating gonococcal strains across US regions over time. Our data are consistent with several previous and recent studies which have indicated that *N. gonorrhoeae* with elevated cephalosporin MICs has emerged in the United States through several distinct lineages via repeated importation from other countries, clonal expansion, extensive recombination events, or a combination of these factors^26,28,29,59,60^. We identified five distinct lineages of *N. gonorrhoeae* with elevated cephalosporin MICs in the United States that could primarily be attributed to mosaic *penA*-34 and derivative alleles (Fig. 1).

A smaller clade of CFM^em^ isolates in the MLST ST1580 lineage, first described as “cef^RS^ cluster 2” by Grad et al.^26^, but referred to as Clade E in this study, showed evidence of persistence as late as 2012, but appears to be no longer circulating. Why this lineage is no longer spreading is unclear. However, it is possible that a lack of biological fitness, susceptibility to other antimicrobials (e.g., ciprofloxacin and ceftriaxone), successful outbreak control, or a combination of these and other public health interventions may explain a lack of further dissemination.

We found evidence of two emerging CFM^em^ clades with additional resistances to penicillin, tetracycline, and ciprofloxacin^7,14,48,61^. These lineages contained isolates with the MLST ST14283 and ST7363 (Clade D, *n* = 6, 2012–2017) and MLST ST1600 and ST1579 (Clade G, *n* = 6, 2014–2017) designation in addition to mosaic *penA* alleles (*penA*-10 and *penA*-27, respectively). Notably, the MLST ST7363 lineage among others (e.g., ST1901) have been attributed to the historical emergence and spread of gonococcal strains with mosaic *penA*-10 and reduced susceptibility to cephalosporins, especially cefixime, and other oral ESCs, beginning around the mid-1990s in metropolitan Japanese cities^7,14,24^. The ability to identify these clades at fine resolution highlights the utility of WGS-based phylogenomics when combined with epidemiologic and phenotypic data in unraveling various strain populations. Future studies that incorporate data from this study will reveal if these lineages continue to persist and how they compare to known strains with reduced susceptibility to cephalosporins.

Perhaps our most significant findings came from analysis of Clades A-B, the largest lineage containing ESC^em^ isolates in our dataset. First described as “cef^RS^ cluster 1” by Grad et al.^26^, this lineage showed evidence of continuing persistence across the United States, particularly on the Westcoast, and was predominantly within MSM networks, with a limited number of introductions into heterosexual networks^26^. Based on our phylogenomic analysis of genetically similar MLST ST1901 isolates from 1992 to 2017, we provide additional evidence that these strains share a common ancestor, and are likely descended from the clonal spread of strains originating from East Asia^7,10,14,24^. We estimate that a common ancestor of the Clade A-B ST1901 lineage may have emerged around the early twentieth century (1925, CI: 1913–1951), and spread into two deeply split lineages. Considering historical trends in global antibiotic usage, our data support other studies that propose a successive acquisition of AMR determinants in these ST1901 strains over time. In fact, the vast majority of all ST1901 strains in our dataset possessed a genomic background associated with resistance to penicillin (widely used between mid-1940s–1970s), tetracycline (1940s–1960s; mediated by *rpsJ* V57M mutations and *tetM* plasmid), and fluoroquinolones (widely used in the 1980s; mediated by *gyraA* S91F, D95A, D95G; *parC* S87R mutations)^10^. Evidence appears to support the hypothesis that the ST1901 strains acquired the penicillin, tetracycline, and the ciprofloxacin resistance phenotype first, before acquiring reduced susceptibility to ESCs^20–22^.

With regard to Clade AB1 strains with mosaic *penA*-34 and derivative alleles, we estimate that their most recent common ancestor emerged around the mid-1980s (1985; CI: 1980–1990, Fig. 2/Node # 7). This includes the oldest isolate in our dataset (34789_MU_NG5) from 1992, which harbors a *penA*-10 allele. It has been proposed that *penA*-10 originated (April 1986–August 1995) from a recombination event in a *penA*-5 background, and that the origin of *penA-34* is the result of an additional recombination event (May 1990–July 1999) from a *penA*-1 donor strain into a *penA*-10 background^62^. When examining several of the Clade AB1 lineages with mosaic *penA*-34 or derivatives on an individual basis, most appear to have emerged around the 1990s (1990; CI: 1986–1994, Fig. 2/Node #9). These results largely recapitulate those of the recent Osnes et al.^63^ study, albeit with slight differences in timing (hereafter we report our tMRCAs and CIs), which suggested two waves of expansion of ST1901 strains out of East Asia: an initial Wave 1 [1970s (1964–1979)], followed by reintroductions in East Asia from Europe and North America, and finally a larger global dispersal of the dominant ST1901 lineage between the 1980s and 1990s [Wave 2; 1987(1983–1992)].

Our current study has several limitations. Notably, samples from the GISP sentinel surveillance system include those from only male patients with symptomatic urethritis at select STD clinics across the United States. Therefore, bias is introduced by not only the populations that visit the clinics but also the exclusion of women who are often asymptomatic, asymptomatic men, extragenital sites of infection, and thus GISP may not fully represent all gonorrhea in the United States. Second, the final sequenced dataset contains most, but not all isolates with elevated cefixime or ceftriaxone MICs. Considering these limitations, findings from our study suggest that patterns of AMR may be generalizable for the populations collected from in the United States between the years 2005 and 2017.

Overall, our analyses support previous studies that have suggested reduced susceptibility to ESCs has emerged and spread independently between and within multiple sexual networks in the United States. We show that the acquisition of cephalosporin-associated AMR has occurred primarily through extensive recombination events, limited de novo mutations, and possible importation from other countries. Given that ceftriaxone is the cornerstone of current therapies and there are few alternatives, it is important to continue carefully monitoring cephalosporin MICs, facilitate programs aimed at increased screening and reducing *N. gonorrhoeae* transmission, particularly in the populations most affected. Finally, our study underscores the continuing need for drugs with novel mechanisms, and diagnostic point-of-care tests to rapidly screen gonococcal infections to identify susceptibility and provide personalized patient care.

## Methods

### Isolate selection

Antimicrobial susceptibilities were determined using the agar dilution method, with some further analyzed by ETEST for end-pointing (bioMerieux, France)^29^. Because the Clinical and Laboratory Standards Institute has not established resistance breakpoints for cefixime or ceftriaxone and only state susceptibility (≤0.25 µg/mL for both antimicrobials), we defined elevated MICs as follows: elevated cefixime (CFM^em^; MIC ≥0.25 µg/mL), ceftriaxone (CRO^em^; MIC ≥0.125 µg/mL), and isolates with elevated MICs to both ESCs (ESC^em^). International isolates with high-level cefixime and/or ceftriaxone MICs were included as references (WGS data were downloaded manually or using SRA toolkit v2.8; http://ncbi.github.io/sra-tools/). Strains from Japan (FC428, FC460) and Australia (A7846, A7536) are available under BioProject PRJNA416507, and a strain from Canada (47707) is available under BioProject PRJNA415047^32,45^. Three strains available in the World Health Organization [WHO] reference panel as WHO-X (H041, Japan),WHO-Y (F89, France), and WHO-Z (A8806, Australia) were also included for comparison^64^. A summary of elevated MIC frequency, MLST STs, and intra-clade SNP distances observed in major clades of interest is presented in Table 1.

Between January 1, 2005 and December 31, 2017, 72,807 urethral gonococcal isolates were collected from symptomatic men through GISP. Overall, 434 GISP isolates were identified with elevated MICs to ESCs, including 293 isolates (68%) that exhibited elevated cefixime MICs, 48 (11%) isolates that exhibited elevated ceftriaxone MICs, and 93 (21%) isolates that exhibited elevated MICs to both ESCs. A convenience sampling of the 72,807 resulted in a total of only 813 isolates being sequenced. Of the 434 GISP isolates with elevated MICs to ESCs, 355 were included in the sequenced isolates with 251 isolates having only elevated cefixime MICs, 23 isolates having only elevated ceftriaxone MICs and 81 isolates having elevated MICs to cefixime and ceftriaxone. Collectively, this resulted in 332 isolates (41%) with elevated cefixime MICs and 104 isolates (13%) with elevated ceftriaxone MICs being included in the dataset. All gonococcal isolates with elevated cephalosporin MICs were matched as closely as possible to susceptible isolates with sequencing data, based on similar region and year. Isolates that were not sequenced by CDC or its partners were missing at random, and unavailable in freezer inventories for unknown reasons. Furthermore, isolates were distributed throughout various US Department of HHS regions, although the majority were overrepresented from the western regions in overall accordance with GISP design (54%, HHS:8, *n* = 11; HHS:9, *n* = 372; HHS:10, *n* = 54) and midwestern region (19%, HHS:5, *n* = 126; HHS:7, *n* = 31)^29^. The GISP isolates in the sequenced dataset were collected from male patients with varying sex of sex partners, including those with female only partners (MSW, 42%), male only partners (MSM, 49%), both male and female partners (MSMW, 8%), and unknown (1%). Additional details on the full dataset are provided in Supplementary Data 1 and a Microreact project: https://microreact.org/project/ESC2005-2017JT^65^.

### Whole genome sequencing and bioinformatics analyses

All 813 *N. gonorrhoeae* isolates from GISP were sequenced on an Illumina Miseq sequencer at the CDC core sequencing facility (Atlanta, GA) or at either the Maryland, Tennessee, Texas, or Washington State Public Health Laboratories, which are all part of the Antibiotic Resistance Laboratory Network regional laboratories. All sequencing reads were initially screened using Kraken v0.10.5, to identify reads assigned to bacterial strains other than *N. gonorrhoeae*. Samples with more than 10% of raw reads assigned to *Neisseria meningitidis* were considered contaminated. In addition, reads were trimmed using CutAdapt v1.8.3 to remove adaptor sequences and bases using a minimum quality cutoff of <Q30 and a minimum length cutoff of 19. Illumina reads were de novo assembled using Spades v3.9.0 under default parameters^66^. For phylogenetic analysis, a full-length whole genome alignment was generated using snippy v4.3.8 (https://github.com/tseemann/snippy) with FA19 (GenBank accession CP012026.1 set as the reference)^67^. This full-length whole genome alignment was used as an input to Gubbins v2.3.1 for identifying and filtering regions of homologous recombination. The resulting alignment, containing only the polymorphisms present in the non-recombinant regions, was used as input for RAxML v8.2.9 under the GTR + GAMMA model of nucleotide substitution with a majority-rule consensus convergence criterion, to reconstruct an ascertainment bias corrected (Stamatakis method) ML phylogeny^42,68^. Analysis of population structure within the ML phylogeny was conducted using Fastbaps v1.0 to identify major clusters under default parameters^69^. Pairwise SNP distances between isolate genomes were calculated using Snp-dists v0.4 (https://github.com/tseemann/snp-dists) using the recombination removed alignment from Gubbins as input. Determination of AMR genotypes was determined using a combination of several tools including an in-house AMR profiler or tblast alignment of contigs against a reference genes extracted from FA19 in Geneious Prime v2019.2.3 (https://www.geneious.com/prime/). MLST and NG-MAST schemes were determined by StringMLST v0.6.3 and NGMASTER v0.5.5, respectively^70,71^. WGS data were also screened against the NG-STAR v2.0 database using pyngSTar (https://github.com/leosanbu/pyngSTar)^72^.

To generate a more detailed understanding of potential introductions and geographic spread of isolates with elevated cephalosporin MICs and mosaic *penA* alleles, we performed a timed-scaled phylogenetic analysis using BEAST (Bayesian Evolutionary Analysis Sampling Trees) v1.8.4. In total we examined 805 gonococcal genomes collected over multiple years ranging from 1992 to 2017. We used TempEst v1.5.3 to determine if there was a sufficient temporal signal for molecular clock analysis and estimated the root-to-tip correlation was 0.5258 (*R*^2^ = 0.2765; Supplementary Fig. 4)^73^. The 805 isolates consisted of 340 GISP isolates identified in Clade A-B of this study, and 465 international isolates (MLST ST1901) obtained from PubMLST that were recently placed within the cgMLST cgc400 core genome group no. 3^50^. We confirmed that these isolates clustered together by applying fastbaps to the original ML phylogeny containing the 813 GISP isolates but with the additional 465 international isolates, as not all MLST ST1901 strains in PubMLST share a similar genomic background (Supplementary Fig. 5).

Initially, we used ABACAS2 to create a concatenated and ordered “pseudochromosome” of isolate 34789_MU_NG5 (Philippines, 1992; accession SRR969380) against the WHO-Y reference genome (F89, MLST ST1901; accession LT592161)^74^. We removed all the Ns that ABACAS2 adds to the overlapping regions where any two contigs are joined, and used this updated pseudochromosome as the reference input for snippy v4.3.8 (https://github.com/tseemann/snippy) to generate a whole genome alignment of the abovementioned MLST ST1901 strains. Following the identification and masking of recombinant SNPs in Gubbins, the resulting alignment was used as the input for BEAST v1.8.4^75^. An ascertainment bias correction step was included by providing the count of the monomorphic sites for each nucleotide in the reference within the BEAST input XML file generated in BEAUti v1.8.4^75^. A HKY (Hasegawa, Kishino, and Yan) substitution model provided by ModelTest-NG v0.1.6, a coalescent model with constant population size, and both strict and uncorrelated relaxed clock models with an exponential distribution as the prior for the clock rate were used for the BEAST analysis^76^. Eight independent chains of 100 million steps were run for clock model, with samples taken every 1000 steps to ensure good mixing. The results were compared using Tracer v1.7.1 to confirm convergence, and the maximum clade credibility trees were generated using Tree Annotator, which are both provided with the BEAST v1.8.4 package.

### Statistical analyses and data visualization

Fisher’s exact or *χ*^2^ test were used to determine the association between mutational patterns, STs, sex of sex partner, or clades with respect to elevated MICs respective of each antibiotic. *P* values and 95% confidence intervals were used to determine statistical significance. A Bonferroni correction was applied for multiple tests^77^. Phylogenetic trees were initially visualized using iToL v6, then redrawn using phandango v1.3.0 (https://github.com/jameshadfield/phandango) and Microreact v5.93.0^65,78,79^. The ggtree v2.5.1 R package (https://github.com/YuLab-SMU/ggtree) was used to visualize all BEAST generated time-scaled phylogenies^80^.

### Reporting summary

Further information on research design is available in the Nature Research Reporting Summary linked to this article.

## Supplementary information

Supplementary Information

Descriptions of Additional Supplementary Files

Supplementary Data 1

Supplementary Data 2

Reporting Summary

## Data Availability

Raw sequencing data and genome assemblies for isolates in this study are included as either BioProject (NCBI Sequence Read Archive or European Nucleotide Archive accession codes, or PubMLST identification numbers) (Supplementary Data 1 and 2). Additional data supporting the findings of this study are available in text and Supplementary information files.
