# Supplementary material for: Phylogenomic analysis reveals persistence of gonococcal strains with reduced-susceptibility to extended-spectrum cephalosporins and mosaic penA-34
Source: Nat Commun. 2021 Jun 21;12:3801. doi: 10.1038/s41467-021-24072-1 (PMC8217231; doi:10.1038/s41467-021-24072-1)
Supplement: Supplementary file 1 — Supplementary Information [file 41467_2021_24072_MOESM1_ESM.pdf]

Supplementary Information

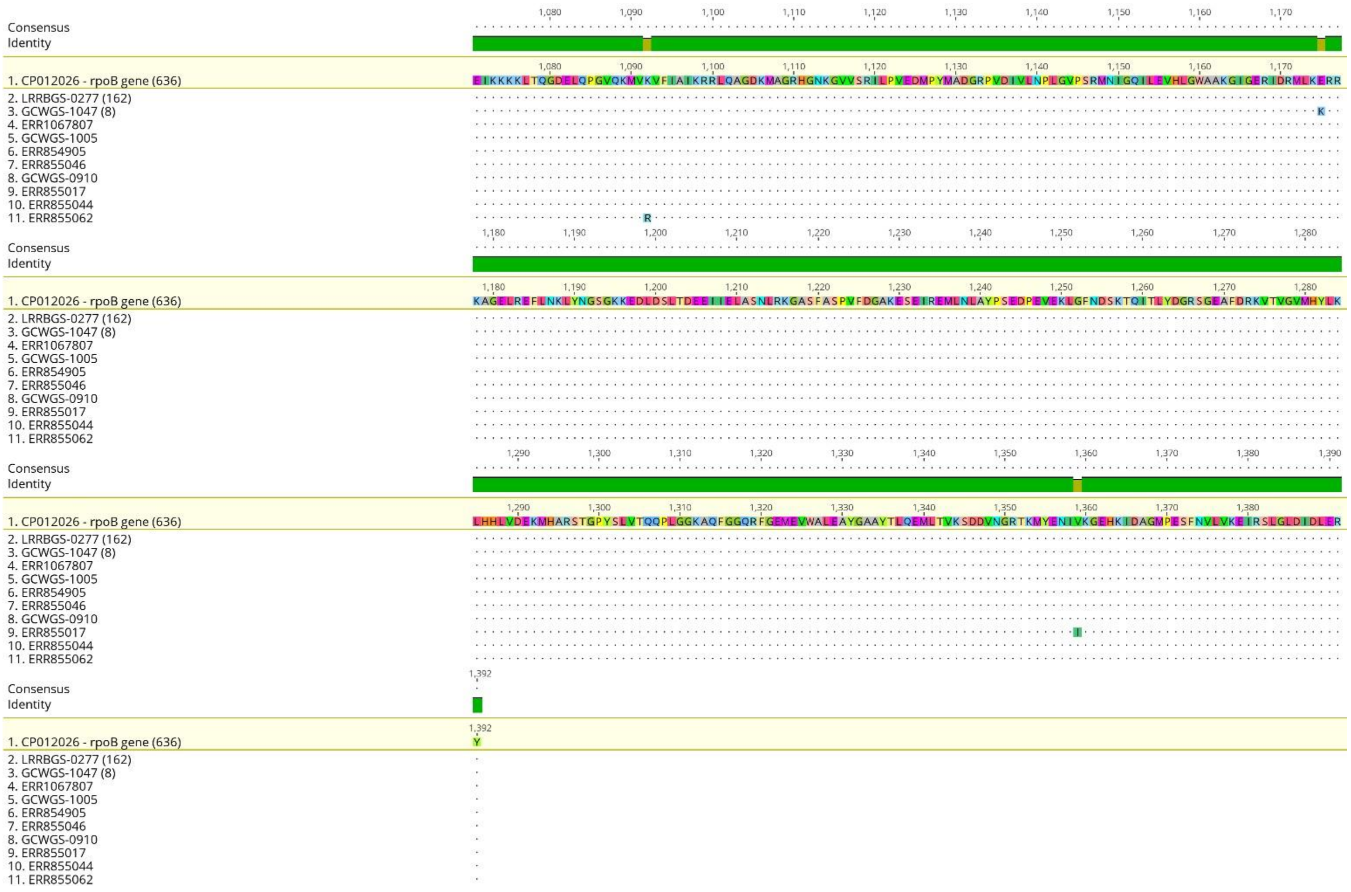

Supplementary Figure 1. Amino acid sequence alignment of all extracted *rpoB* genes from whole genome sequence assemblies. The strain FA19 (accession no. CP012026.1) was used as the reference. Representative isolates are listed in numbered rows, with the number of isolates with an identical allele indicated in parentheses.

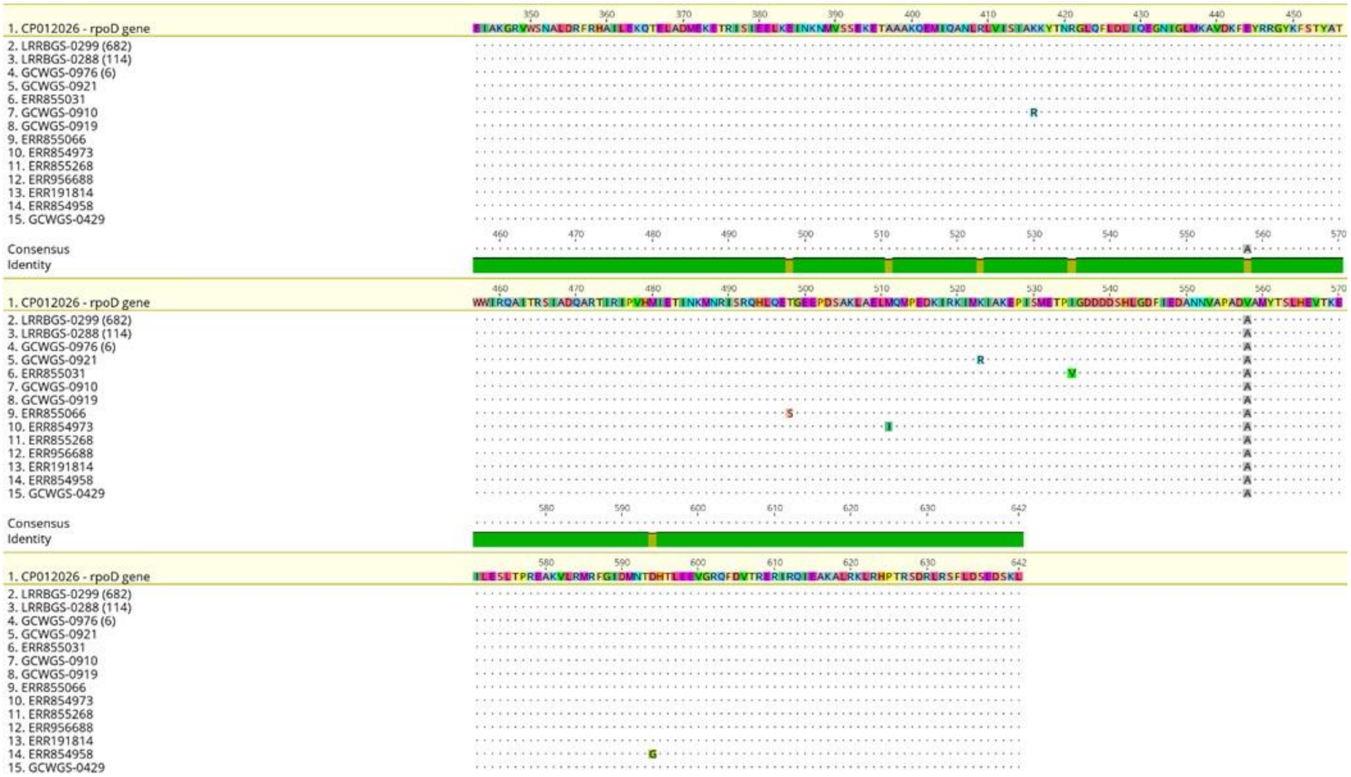

Supplementary Figure 2. Amino acid sequence alignment of all extracted *rpoD* genes from whole genome sequence assemblies. The strain FA19 (accession no. CP012026.1) was used as the reference. Representative isolates are listed in numbered rows, with the number of isolates with an identical allele indicated in parentheses.

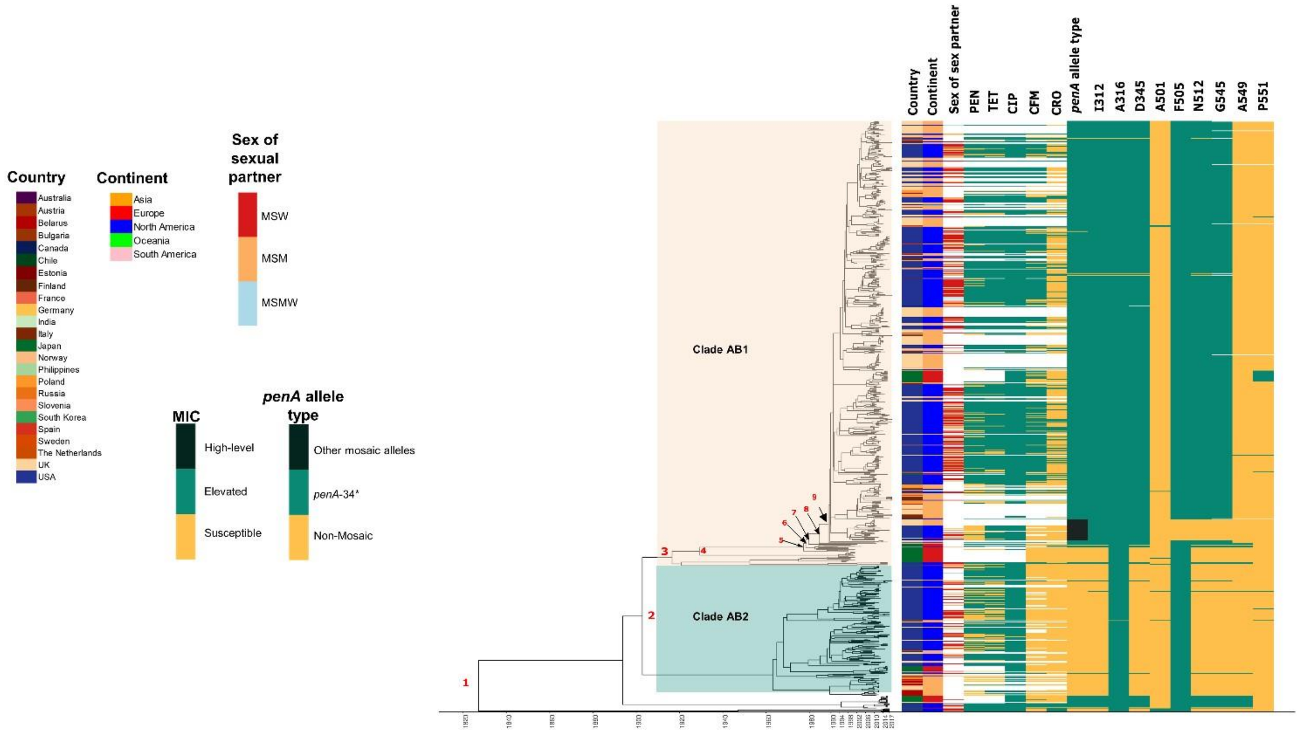

Supplementary Figure 3. Time-scaled (relaxed model) BEAST phylogeny of 805 isolates, including 340 GISP isolates identified in Clade A-B of this study and 465 international MLST ST1901 strains. CladeAB1 (light orange) and Clade-AB2 (light green) represent the divergent sub-lineages which we identified in the phylogeny. The oldest sequenced isolate is from the Philippines collected in 1992. Sex of sex partners are colored [MSW – men that have sex with women (red), MSM – men that have sex with men (light orange), MSMW – men that have sex with men and women (light blue). Antimicrobial susceptibility is based on the minimum inhibitory concentration (MIC) to each respective antibiotic [penicillin (PEN), tetracycline (TET), ciprofloxacin (CIP), cefixime (CFM), ceftriaxone (CRO)] and is shown with colors: susceptible (light orange), elevated MIC range (teal), or high-level MICs (dark green). Alleles for *penA* are colored based on their amino acid sequence homology to *penA*-34 (≥98% is a derivative allele; teal), differing mosaic *penA* allele (dark green), or absence of a mosaic *penA* allele. Amino acid mutations in PBP2 associated with elevated cephalosporin MICs are shown at the top of figure. The bottom scale bar represents the time to the most recent common ancestor (tMRCA) of the internal node of the phylogenetic tree. Numbers highlighted in red are the internal ancestral nodes of the time-scaled phylogenetic tree and represent estimated years and credibility intervals based on tMRCA: 1) Root; 1828 [1746 - 1856], 2) 1901 [1865 - 1933], 3) 1908 [1870 - 1935], 4) 1931 [1885 - 1964], 5) 1970 [1959 - 1984], 6) 1971 [1962 - 1980], 7) 1977 [1966 - 1988], 8) 1981 [1976 - 1992], 9) 1987 [1981-1997].

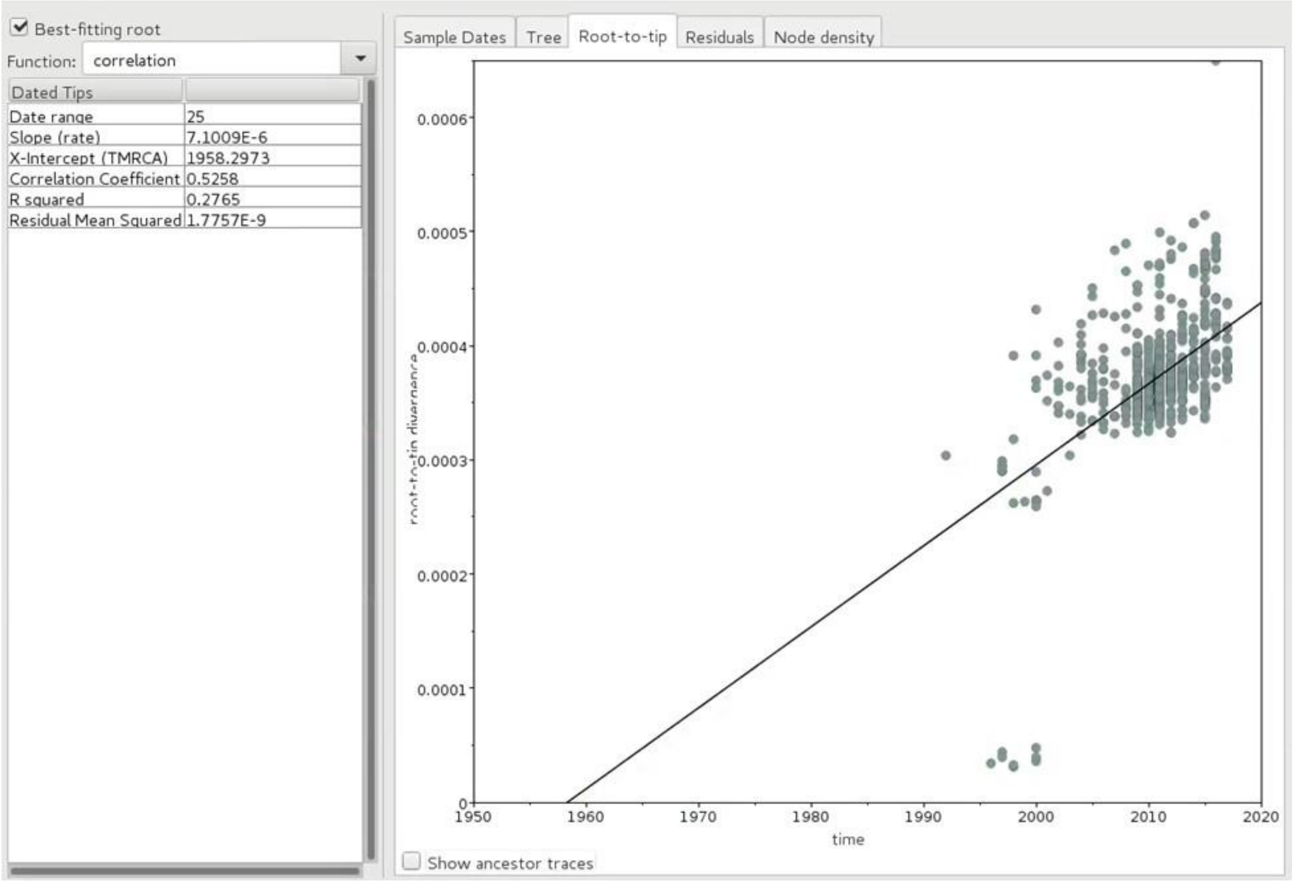

Supplementary Figure 4. Figure showing the Root-to-tip regression analysis estimated using TempEst for the final dataset.

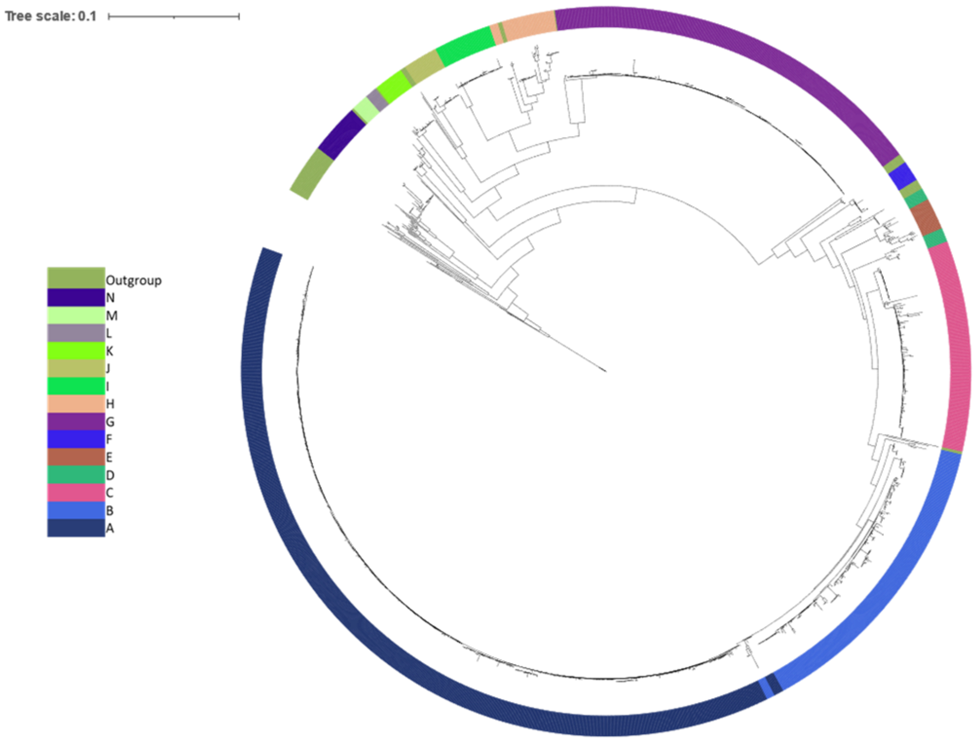

Supplementary Figure 5. Maximum likelihood tree based on core-genome SNPs of 1,587 *Neisseria gonorrhoeae* strains collected globally from 1992 to 2017. Fastbaps was used to identify MLST ST1901 lineages with reduced susceptibility to extended spectrum cephalosporins as shown in Clade A (dark blue) and Clade B (royal blue).

Table 1. Parameter and effective sample sizes (ESS) estimates for the BEAST analysis with strict clock model.

Statistics	Mean estimate	Effective Sample Size (ESS)
posterior	-2.041×10^{6}	234
prior	-6198.889	204
likelihood	-2.034×10^{6}	228
treeModel rootHeight	202.87	222
constant.popSize	815.689	228
kappa	6.175	47337
frequencies1	0.228	4.113×10^{5}
frequencies2	0.266	4.057×10^{5}
frequencies3	0.272	4.065×10^{5}
frequencies4	0.235	4.098×10^{5}
clock.rate	1.853×10^{-6}	206
meanRate	1.853×10^{-6}	206
treeLikelihood	-2.034×10^{6}	228
coalescent	-6189.013	204

Table 2. Parameter and effective sample sizes (ESS) estimates for the BEAST analysis with relaxed clock model.

Statistics	Mean estimate	Effective Sample Size (ESS)
posterior	-2.05×10^{6}	203
prior	-17737.781	215
likelihood	-2.032×10^{6}	202
treeModel rootHeight	171.177	268
constant.popSize	537.557	251
kappa	6.188	11216
frequencies1	0.228	3.305×10^{5}
frequencies2	0.266	3.137×10^{5}
frequencies3	0.272	3.197×10^{5}
frequencies4	0.235	3.185×10^{5}
ucld.mean	3.071×10^{-6}	231
ucld.stddev	1.007	601
meanRate	2.378×10^{-6}	203
coefficientOfVariation	1.182	318
covariance	3.441×10^{-2}	567
treeLikelihood	-2.032×10^{6}	202
branchRates	-11871.456	constant
coalescent	-5854.94	215

Table 3. Estimated years of the origins of few selected ancestral nodes using the strict and relaxed clock models implemented in BEAST. Estimated years are based on the most recent common ancestor (MRCA). Note that the credibility intervals are overlapping for all nodes for the analysis using the strict and relaxed model, while the latter model has longer credibility intervals than the former model. *The internal node numbers are shown in the both of the timescaled phylogenetic tree reconstructed using the strict (**Figure 2**) and relaxed clock model (**Supplementary figure 3**)

**Internal Node #'s***	**Strict clock model** (substitution rate = 1.8215 x 10^{-6} substitutions per site per year; 95% HPD; 1.62323 x 10^{-6} to 2.069 x 10^{-6})	**Relaxed clock model** (substitution rate = 2.3638 x 10^{-6} substitutions per site per year; 95% HPD; 2.035 x 10^{-6} to 3.1145 x 10^{-6})
	**Year of the MRCA (Credibility interval)**	**Year of the MRCA (Credibility interval)**
1 (Root)	1818 [1781 - 1865]	1828 [1746 - 1856]
2	1925 [1913 - 1951]	1901 [1865 - 1933]
3	1944 [1935 - 1953]	1908 [1870 - 1935]
4	1952 [1942 - 1964]	1931 [1885 - 1964]
5	1970 [1964 - 1977]	1970 [1959 - 1984]
6	1972 [1966 -1979]	1971 [1962 - 1980]
7	1985 [1980 - 1990]	1977 [1966 - 1988]
8	1987 [1983 - 1992]	1981 [1976 - 1992]
9	1990 [1986 - 1994]	1987 [1981 – 1997]
