# Supplementary material for: Phylogenomic analysis reveals persistence of gonococcal strains with reduced-susceptibility to extended-spectrum cephalosporins and mosaic penA-34
Source: Nat Commun. 2021 Jun 21;12:3801. doi: 10.1038/s41467-021-24072-1 (PMC8217231; doi:10.1038/s41467-021-24072-1)
Supplement: Supplementary file 2 — Descriptions of Additional Supplementary Files [file 41467_2021_24072_MOESM2_ESM.pdf]

Descriptions of Additional Supplementary Files

Supplementary Data 1
Description: Table containing information on sequencing identifiers of GISP isolates collected from 2005 to 2017, based on the Sequence Read Archive (SRA), collection year, sex of sex partner [MSM – men that have sex with men, MSMW – men that have sex with men and women, MSW – men that have sex with women], Health and Human Services (HHS) region), multi-locus sequence type (MLST), Neisseria gonorrhoeae multi-antigen sequence type (NGMAST), Neisseria gonorrhoeae sequence typing for antimicrobial resistance (NG-STAR), core genome sequence type for Neisseria gonorrhoeae (cgST), Neisseria gonorrhoeae core genome group 400 (Ng_cgc_c400). For mutations, a nucleotide deletion is represented by "DEL", wildtype gene is represented by "WT", and NF represents that a mutation was not found at position due to a partially assembled gene.

Supplementary Data 2
Description: Table containing information on sequence identifiers of all Clade AB isolates examined for time-scaled phylogenetic analyses. Isolates are listed based on PubMLST identification numbers and/or sequence read archive (SRA) accession numbers. Sex of sex partner identifiers are as follows: men that have sex with men (MSM), men that have sex with men and women (MSMW), men that have sex with women (MSW). Antimicrobial suscepetiblity is based on minimum inhibitory concentrations (MIC) to penicillin (PEN), tetracycline (TET),), ciprofloxacin (CIP), cefixime (CFM), and ceftriaxone (CRO). Amino acid (aa) mutations are also provided and are described as wild-type (WT) or mutation at specific positions in the penA gene.
