# Supplementary material for: Phylogenomic analysis reveals persistence of gonococcal strains with reduced-susceptibility to extended-spectrum cephalosporins and mosaic penA-34
Source: Nat Commun. 2021 Jun 21;12:3801. doi: 10.1038/s41467-021-24072-1 (PMC8217231; doi:10.1038/s41467-021-24072-1)
Supplement: Supplementary file 5 — Reporting Summary [file 41467_2021_24072_MOESM5_ESM.pdf]

nature research

Corresponding author(s): Jesse C. Thomas, IV

Last updated by author(s): 05/19/2021

Reporting Summary

Nature Research wishes to improve the reproducibility of the work that we publish. This form provides structure for consistency and transparency in reporting. For further information on Nature Research policies, see our Editorial Policies and the Editorial Policy Checklist.

Statistics

For all statistical analyses, confirm that the following items are present in the figure legend, table legend, main text, or Methods section.

n/a	Confirmed	
☐	☒	The exact sample size (n) for each experimental group/condition, given as a discrete number and unit of measurement
☒	☐	A statement on whether measurements were taken from distinct samples or whether the same sample was measured repeatedly
☐	☒	The statistical test(s) used AND whether they are one- or two-sided<br>*Only common tests should be described solely by name; describe more complex techniques in the Methods section.*
☒	☐	A description of all covariates tested
☐	☒	A description of any assumptions or corrections, such as tests of normality and adjustment for multiple comparisons
☐	☒	A full description of the statistical parameters including central tendency (e.g. means) or other basic estimates (e.g. regression coefficient) AND variation (e.g. standard deviation) or associated estimates of uncertainty (e.g. confidence intervals)
☐	☒	For null hypothesis testing, the test statistic (e.g. F, t, r) with confidence intervals, effect sizes, degrees of freedom and P value noted<br>*Give P values as exact values whenever suitable.*
☐	☒	For Bayesian analysis, information on the choice of priors and Markov chain Monte Carlo settings
☐	☒	For hierarchical and complex designs, identification of the appropriate level for tests and full reporting of outcomes
☒	☐	Estimates of effect sizes (e.g. Cohen's d, Pearson's r), indicating how they were calculated

Our web collection on statistics for biologists contains articles on many of the points above.

Software and code

Policy information about availability of computer code

Data collection	SRA toolkit 2.8 for fastq or assembly collection
Data analysis	microreact v5.93.0: https://microreact.org<br>CutAdapt v1.8.3<br>snippy v4.3.8 (https://github.com/tseemann/snippy)<br>Gubbins v2.3.1<br>RaxML v8.2.9<br>Fastbaps v1.0<br>Snp-dists v0.4 (https://github.com/tseemann/snp-dists)<br>Geneious Prime v2019.2.3 (https://www.geneious.com/prime/)<br>StringMLST v0.6.3<br>NGMASTER v0.5.5<br>NG-STAR v2.0 database using pyngSTar (https://github.com/leosanbu/pyngSTar)<br>Beast v1.8.4<br>Tracer v1.7.1<br>TempEst v1.5.3<br>pubMLST.org /bigsdb for cgMLST analysis<br>ABACAS2 (https://github.com/sangerpathogens/ABACAS2)<br>BEAUti (part of BEAST v1.8.4)<br>ModelTest-NG v0.1.6<br>Tree Annotator v1.8.4 (part of BEAST v1.8.4)<br>iTOL v6

ggtree v2.5.1
phandango v1.3.0 (https://github.com/jameshadfield/phandango)
Kraken v0.10.5
Spades v3.9.0

For manuscripts utilizing custom algorithms or software that are central to the research but not yet described in published literature, software must be made available to editors and reviewers. We strongly encourage code deposition in a community repository (e.g. GitHub). See the Nature Research guidelines for submitting code & software for further information.

Data

Policy information about availability of data

All manuscripts must include a data availability statement. This statement should provide the following information, where applicable:
- Accession codes, unique identifiers, or web links for publicly available datasets
- A list of figures that have associated raw data
- A description of any restrictions on data availability

Raw sequencing data, genome assemblies for isolates in this study are included as either BioProject (NCBI Sequence Read Archive [https://www.ncbi.nlm.nih.gov/sra] or European Nucleotide Archive [https://www.ebi.ac.uk/ena/browser/home]) accession codes, or PubMLST identification numbers (Supplementary Table S2). Additional data supporting the findings of this study are available in text and Supplementary Data files.

Field-specific reporting

Please select the one below that is the best fit for your research. If you are not sure, read the appropriate sections before making your selection.

☒ Life sciences ☐ Behavioural & social sciences ☐ Ecological, evolutionary & environmental sciences

For a reference copy of the document with all sections, see nature.com/documents/nr-reporting-summary-flat.pdf

Life sciences study design

All studies must disclose on these points even when the disclosure is negative.

Sample size	Sample size was determined on the available sequences that matched MIC criteria.
Data exclusions	We have no data exclusions to report.
Replication	All results can be replicated using the available sequences and the various scripts/software packages described in order of usage in the manuscript
Randomization	Isolates were not chose at random. They were selected by presence of an elevated MIC to cefixime/ceftriaxone which is detailed in the manuscript, and then matched with susceptible isolates from the same time period/region as close as possible
Blinding	This is N/A. Blinding was not necessary since this is a phylogenomic analysis using surveillance data. As part of sentinel surveillance GISP does not receive any personal identifying information that could be used to identify a particular patient visiting an STD clinic.

Reporting for specific materials, systems and methods

We require information from authors about some types of materials, experimental systems and methods used in many studies. Here, indicate whether each material, system or method listed is relevant to your study. If you are not sure if a list item applies to your research, read the appropriate section before selecting a response.

Materials & experimental systems

n/a	Involved in the study	
☒	☐	Antibodies
☒	☐	Eukaryotic cell lines
☒	☐	Palaeontology and archaeology
☒	☐	Animals and other organisms
☒	☐	Human research participants
☒	☐	Clinical data
☒	☐	Dual use research of concern

Methods

n/a	Involved in the study	
☒	☐	ChIP-seq
☒	☐	Flow cytometry
☒	☐	MRI-based neuroimaging
